# An Improved PSO Algorithm for Generating Protective SNP Barcodes in Breast Cancer

**DOI:** 10.1371/journal.pone.0037018

**Published:** 2012-05-18

**Authors:** Li-Yeh Chuang, Yu-Da Lin, Hsueh-Wei Chang, Cheng-Hong Yang

**Affiliations:** 1 Department of Chemical Engineering and Institute of Biotechnology and Chemical Engineering, I-Shou University, Kaohsiung, Taiwan; 2 Department of Electronic Engineering, National Kaohsiung University of Applied Sciences, Kaohsiung, Taiwan; 3 Department of Biomedical Science and Environmental Biology, Center of Excellence for Environmental Medicine, Cancer Center, Kaohsiung Medical University Hospital, Kaohsiung Medical University, Kaohsiung, Taiwan; University of Texas School of Public Health, United States of America

## Abstract

**Background:**

Possible single nucleotide polymorphism (SNP) interactions in breast cancer are usually not investigated in genome-wide association studies. Previously, we proposed a particle swarm optimization (PSO) method to compute these kinds of SNP interactions. However, this PSO does not guarantee to find the best result in every implement, especially when high-dimensional data is investigated for SNP–SNP interactions.

**Methodology/Principal Findings:**

In this study, we propose IPSO algorithm to improve the reliability of PSO for the identification of the best protective SNP barcodes (SNP combinations and genotypes with maximum difference between cases and controls) associated with breast cancer. SNP barcodes containing different numbers of SNPs were computed. The top five SNP barcode results are retained for computing the next SNP barcode with a one-SNP-increase for each processing step. Based on the simulated data for 23 SNPs of six steroid hormone metabolisms and signalling-related genes, the performance of our proposed IPSO algorithm is evaluated. Among 23 SNPs, 13 SNPs displayed significant odds ratio (*OR*) values (1.268 to 0.848; *p*<0.05) for breast cancer. Based on IPSO algorithm, the jointed effect in terms of SNP barcodes with two to seven SNPs show significantly decreasing *OR* values (0.84 to 0.57; *p*<0.05 to 0.001). Using PSO algorithm, two to four SNPs show significantly decreasing *OR* values (0.84 to 0.77; *p*<0.05 to 0.001). Based on the results of 20 simulations, medians of the maximum differences for each SNP barcode generated by IPSO are higher than by PSO. The interquartile ranges of the boxplot, as well as the upper and lower hinges for each n-SNP barcode (n = 3∼10) are more narrow in IPSO than in PSO, suggesting that IPSO is highly reliable for SNP barcode identification.

**Conclusions/Significance:**

Overall, the proposed IPSO algorithm is robust to provide exact identification of the best protective SNP barcodes for breast cancer.

## Introduction

Genome-wide association studies (GWAS) can identify several highly robust and statistically significant single nucleotide polymorphisms (SNPs) associated with breast cancer susceptibility [1]–[6]. The associations for genotype frequencies of case and control data have significant impacts on the disease susceptibility. Although GWASs provide representative SNPs from the entire genome, many SNPs with a low or marginal significance are frequently excluded to effectively retrieve highly significant and representative tagSNPs.

A steroid hormone metabolism and signalling-related genes are implicated in the pathogenesis of breast cancer [7]–[12]. Several single nucleotide polymorphism (SNP) association studies involved these genes, such as the estrogen receptor 1 (ESR1), steroid sulfatase (microsomal), isozyme S (STS), cytochrome P450, family 19, subfamily A, polypeptide 1 (CYP19A1), progesterone receptor (PGR), catechol-O-methyltransferase (COMT), and sex hormone-binding globulin (SHBG), have all been reported in these studies [13]–[15].

Many studies hypothesize that the cancer or disease risk is associated with the co-occurrence of SNPs displaying a jointed effect [16]–[22]. In recent breast cancer association studies, further evidence for SNP-SNP interactions has been identified, such as the SNP-SNP interactions of genes related to DNA repair [23], [24], chemokine ligand-receptor interactions [25], and estrogen-response gene [6]. However, the possible SNP-SNP interactions between these hormone metabolisms and signalling-related genes have hardly been addressed. This is in part due to the computationally challenging nature of association studies with multiple SNP candidates.

Currently, analysis of SNP-SNP interactions remains challenge because of the complex combination of data with huge SNPs. Many possible combinations of alleles in SNP-SNP interactions are generated when multiple SNPs are evaluated simultaneously. Mathematically, the possible combinations of SNP interactions between cases and controls is estimated to be C(N,M)*3^M^ = N!/[M! (N-M)!]*3^M^, where *N* is the number of SNPs or factors, and *M* is the selected prediction number of SNPs. Many artificial intelligence methods have been proposed to compute the association of genotype frequencies of case and control data. They were demonstrated to be effective in reducing the number of search items among a greater number of SNP combinations, such as multifactor dimensionality reduction (MDR) [26], [27], polymorphism interaction analysis (PIA) [28], support vector machine (SVM) [29], particle swarm optimization (PSO) [30], and genetic algorithm (GA) [31]. In general, MDR provides many useful features but tends to yield false positive and negative errors when the case/control ratio in a combination of genotypes is similar to the ratio in the entire data set [32]. The PSO and GA methods have the ability to generate relevant SNP combinations in high-dimensional data; however, these methods do not guarantee that every implemented result contains a relevant solution when the dimensionality is very high. This is due to the PSO and GA algorithms using random generator initial values and a set number of iterations. Accordingly, the improved algorithms for solving this complex interaction problem are essential.

Here, we develop an improved PSO algorithm called IPSO that improves the reliability of traditional PSO. This improvement is based on the population initialization step during the PSO process, i.e., keeping good solutions and improving always the concept of best solution during the process; this conservation of superior results yields better solutions for high-order SNP-SNP interactions. We systematically evaluated the joint effects of 23 SNP combinations of six steroid hormone metabolisms and signalling genes involved in breast carcinogenesis. The SNP barcodes generated by the IPSO algorithm were statistically evaluated by the odds ratio and risk ratio to predict breast cancer susceptibility. The results demonstrate that the proposed IPSO method can identify more relevant SNP barcodes for high-dimensional data sets and improved the reliability of the results in the 20 test runs we conducted.

**Figure 1:**
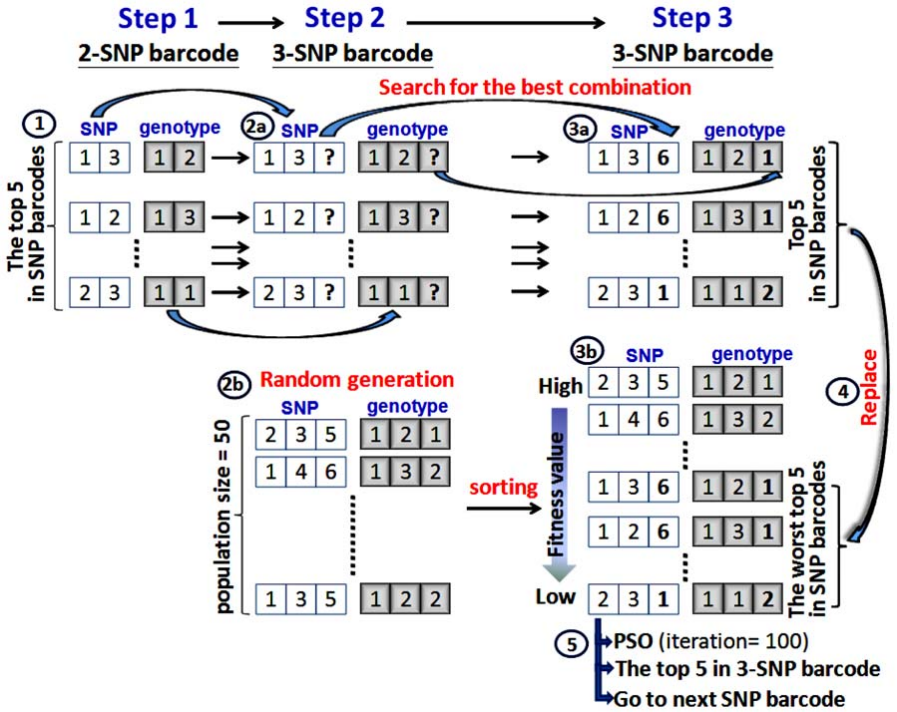
Population initialization using conservation of the best 5 results.

## Methods

### Particle Swarm Optimization

PSO is an efficient evolutionary computation learning algorithm developed by Kennedy and Eberhart [33]. It was originally developed to graphically mimic the unpredictable movement of birds in a flock. The concept of PSO was designed to simulate social behavior based on information exchange, and was designed for practical applications. Within the problem space, each potential solution can be seen as a particle in a swarm. Every particle with a certain velocity can adjust its direction path according to its own flight experience and that of its companions. This superior strategy effectively mines the optimal regions of complex search spaces through the interaction of individuals in a population of particles. The basic elements of PSO are mentioned below:

1. Population: A swarm (population) consisting of *N* particles. Each particle can be regarded as a problem solution in this study.
2. Particle position, 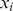: Each candidate solution can be represented by a *D*-dimensional vector; the *i* ^th^ particle can be described as 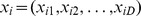, where 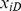 is the position of the *i* ^th^ particle with respect to the *D* ^th^ dimension. Each dimensional vector in particle position is defined by the number of selected SNPs and the corresponding genotypes for the associated SNPs.
3. Particle velocity, 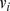: The velocity of the *i* ^th^ particle is represented by 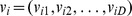, where 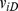 is the velocity of the *i* ^th^ particle with respect to the *D* ^th^ dimension. The new locations of particles are chosen by adding 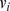 to the coordinate of the particle position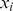; PSO operates this process by adjusting 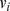. In addition, the velocity of a particle is limited within 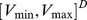.
4. Inertia weight, *w*: The inertia weight is used to control the impact of the previous velocity of a particle on its current velocity. This control parameter affects the trade-off between the exploration and exploitation abilities of the particle.
5. Individual best value, *pbest_i_*: *pbest_i_* is the position of the *i* ^th^ particle with the highest fitness value at a given iteration. It can be currently regarded as a best solution of SNP barcodes so far in terms of *i^th^* particle.
6. Global best value, *gbest*: The best position of all *pbest* particles is called global best. It can be currently regarded as a best solution of SNP barcodes so in all particles.
7. Termination criteria: The process is stopped after the maximum allowed number of iterations is reached.

The PSO algorithm can be divided into four steps within a process period. First, particles are respectively initialized in a population of random solutions. Then each particle finds its own *pbest_i_* by comparing its current fitness to the fitness of its previous position. In a third step, the *gbest* of all the particles in the population is determined. And finally, the PSO algorithm executes a search for optimal solutions by updating the generations. In each generation, the position and velocity of the *i*_th_ particle are updated with *pbest_i_* and *gbest* of the swarm population. The update equations can be formulated as:
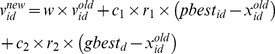
(1)

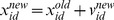
(2)where *w* is the inertia weight. This inertia weight is a positive linear function of time that changes with the generations; *r*_1_ and *r*_2_ are random numbers between (0, 1), and *c*_1_ and *c*_2_ are acceleration constants that control how far a particle moves in a single generation. Velocities 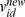 and 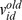, respectively, denote the velocities of the new and old particles; 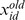 is the current particle position, and 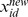 is the updated particle position. The velocity implies the degree to which a particle’s position should be changed at a particular moment in time, so that it can equal that of the global best position, i.e., the velocity of the particle flying toward the best position. To obtain a search solution, the particles’ velocities in each dimension are limited within [*V*_min_, *V*_max_]*^D^*, and the particles’ positions are limited within [*X*_min_, *X*_max_]*^D^*, thus determining the size of the steps the particle is allowed to take through the solution space.

**Table 1:** IPSO pseudo-code.

01: **begin**
02: find the top five 2-SNP barcodes
03: conservation of the best five results Xg≡(*Xg*_1_, *Xg*_2_, *…*, *Xg*_5_)
04: **for** *N* = 3 **to** all numbers of SNP
05: P*_i_*≡(*X_i1_*, *X_i2_*, *…*, *X_ij_*), *i*∈[1.*n*]; *j*∈[1.*d*]
06: µ*X_ij_* ∼*X_j_*(Min, Max), *i*∈[1.*n*]; *j*∈[1.*d*]
07: µ*V_ij_* ∼*V_j_*(Min, Max), *i*∈[1.*n*]; *j*∈[1.*d*]
08: evaluate *P_i_* by Eq. 5, *i*∈[1.*n*]
09: find best Xg in *N-*SNP combinations
10: the worst five *P* are replaced with Xg
11: **repeat PSO:**
12: for each swarm P*_i_*, *i* ∈[1.*n*]
13: *f_i_* ← evaluate *P_i_* by Eq. 3
14: **if** *pbest_i_*<*f_i_* **then**
15: *pbest_i_* ←??*f_i_*; *pbestX_i_* ←*P_i_*
16: **if** *gbest*<*pbest_i_* **then**
17: *gbest* ←*pbest_i_*; *gbestX* ←*pbestX_i_*
18: **end if**
19: **end if**
20: **for** each particle P*_ij_*, *j*∈[1.*d*]
21: *V_ij_*←update *V_ij_* by Eq.122: *X_ij_*←update *X_ij_* by Eq.2
23: **next** *j*
24: **next** *i*
25: **until PSO stopping criterion is met**
26: conservation of the top five results Xg≡(*Xg*_1_, *Xg*_2_, *…*, *Xg*_5_)
27: **end** *N*
28: **end**

### Improved Particle Swarm Optimization

This study proposes a new idea to improve the stability of results obtained with particle swarm optimization. We conserve the best results in the each SNP barcode prediction, which allows us to offer better results for high-order SNP-SNP interactions. The retention of the best results in PSO is very simple and can be done without increasing the computational complexity of the process. The difference between IPSO and PSO is that the proposed new idea is applied in the population initialization step during the PSO process. The IPSO proceeds as follows: The initial population is generated by our strategy and then the fitness values of all individuals in the population are calculated by a fitness function. The particles are repositioned according to their own *pbest* and *gbest* solutions. The procedure is repeated in each successive iteration until the termination conditions are reached.

**Table 2:** Pseudo-code for randomly generated data.

01: **begin**
02: Set size = 5000
03: Set number of genotype = 3
04: Calculate amount of three genotypes
05: **while** (all SNPs are not normalized)
06: Calculate amount of each genotype
07: Calculate numbers of each normalized genotype
08: **for** *n* = 1 to number of genotype
09: Randomly create numbers of each normalized genotype
10: **next** *n*
11: end while
12: **end**

#### Encoding schemes

In IPSO, every particle in a population is associated with a solution group. We define a particle based on the number of selected SNPs, and the genotype associated with the corresponding SNPs; the SNPs cannot be repeatedly selected. The particle encoding can thus be represented by:

where *SNP_i,j_* represents the selected SNP, *Genotype_i,j_* represents the three possible genotypes once *SNP_ i,j_* is selected, *m* represents the size of the population, and *n* represents the number of SNPs selected. Initial particles are randomly generated in this study. For example, let *P* = *(SNP_3,4,8_, Genotype_2,1,3_)*. In this representation of the particle, *SNP_3,4,8_* represents the chosen SNPs (3, 4, 8) and *Genotype_2,1,3_* represents the chosen genotypes (2, 1, 3). In this case, the selected SNPs with their corresponding genotypes are represented as (3, 2), (4, 1), and (8, 3), respectively.

#### Population initialization using conservation of the top five results

The top five results in the 2-SNP barcode and in the n-SNP barcode (n ≧3) are generated differently. For the 2-SNP barcode, we only apply the exhaustive search algorithm to compute and check all possible 2-SNP combinations to give the best five results for all 2-SNP barcodes. To generate the n-SNP barcode (n ≧3), the steps for population initialization are illustrated in Figure 1. To initialize the population, the top five results amongst the previous 2-SNP combinations are used to initialize the population initialization for other numbers of SNP combinations. For example, *(SNP_1,3_, Genotype_1,2_)* is one of the top five 2-SNP barcodes (step 1-1). Subsequently, this 2-SNP barcode is applied to search for the best combination of the 3-SNP barcode with a maximum difference value between the case and control data (step 2-2a); in this example the search result is *(SNP_1,3,i_, Genotype_1,2,j_),* with *i* = {2, 4, 5, 6 … *n | n* representing the number of SNPs} and *j* = {1, 2, 3}. Then, the exhaustive search algorithm is applied to compute and check all possible 3-SNP combinations to find the top five results for all 3-SNP barcodes (step 3-3a). If the exhaustive search algorithm finds the answer to be *i* = 6 and *j* = 1 (the newly added third SNP and the genotype are 6 and 1, respectively), the best 2-SNP barcode *(SNP_1,3_, Genotype_1,2_)* can generate its best 3-SNP barcode *(SNP_1,3,6_, Genotype_1,2,1_).* Similarly, four of the top five 3-SNP barcodes are generated.

**Table 3:** Estimated effect (odds ratio and 95% CI) from individual SNPs of 23 steroid hormone metabolisms and signalling-related genes on the occurrence of breast cancer in patients.

SNP (Gene)^a^	SNPtype	Caseno.	Controlno.	Oddsratio	95% CI	*p*value^e^	SNP (Gene)	SNPtype	Caseno.	Controlno.	Oddsratio	95% CI	*p*value^e^
(Ch/position)^cd^							(Position)						
1. rs6269 (COMT)	1-AA	1694	1769				13. rs9478249 (ESR1)	1-TT	1890	1773			
(22/19949952)	2-AG	2389	2390	1.044	0.955, 1.140	0.337	(6/152199431)	2-TG	2381	2430	0.919	0.843, 1.003	0.056
	3-GG	917	841	1.139	1.013, 1.279	**0.028**		3-GG	729	797	0.858	0.760, 0.969	**0.012**
2. rs4680 (COMT)	1-GG	1308	1377				14. rs1514348 (ESR1)	1-CC	1717	1830			
(22/19951271)	2-GA	2440	2417	1.063	0.966, 1.169	0.211	(6/152182315)	2-CA	2435	2415	1.075	0.985, 1.173	0.107
	3-AA	1252	1206	1.093	0.978, 1.221	0.118		3-AA	851	755	1.201	1.066, 1.354	**0.002**
3. rs10046 (CYP19A1)	1-CC	1434	1430				15. rs532010 (ESR1)	1-TT	1848	1891			
(15/51502986)	2-CT	2411	2497	0.963	0.877, 1.057	0.424	(6/152130918)	2-TC	2377	2422	1.004	0.921, 1.095	0.930
	3-TT	1155	1073	1.073	0.959, 1.201	0.214		3-CC	775	687	1.154	1.021, 1.305	**0.021**
4. rs3020314 (ESR1)	1-CC	2147	2343				16. rs566351 (PGR)	1-TT	2062	2014			
(6/152270672)	2-CT	2280	2164	1.150	1.057, 1.250	**0.001**	(11/100985014)	2-TC	2280	2326	0.957	0.879, 1.043	0.312
	3-TT	573	493	1.268	1.107, 1.453	**0.001**		3-CC	658	660	0.974	0.858, 1.105	0.680
5. rs2234693 (ESR1)	1-TT	1446	1450				17. rs660149 (PGR)	1-CC	2708	2591			
(6/152163335)	2-TC	2480	2524	1.015	0.925, 1.113	0.761	(11/100934314)	2-CG	1927	2042	0.903	0.831, 0.981	**0.016**
	3-CC	1074	1026	1.065	0.961, 1.181	0.232		3-GG	365	367	0.952	0.813, 1.114	0.554
6. rs1543404 (ESR1)	1-TT	1468	1467				18. rs11571171 (PGR)	1-TT	2419	2338			
(6/152428838)	2-TC	2439	2441	0.999	0.910, 1.095	0.981	(11/100974887)	2-TC	2082	2163	0.930	0.856, 1.012	0.091
	3-CC	1093	1092	1.000	0.894, 1.119	1.000		3-CC	499	499	0.967	0.841, 1.110	0.626
7. rs3798577 (ESR1)	1-TT	1413	1406				19. rs500760 (PGR)	1-AA	2888	2994			
(6/152421130)	2-TC	2494	2542	0.976	0.889, 1.072	0.621	(11/100909991)	2-AG	1866	1767	1.095	1.007, 1.190	**0.033**
	3-CC	1093	1052	1.034	0.922, 1.159	0.567		3-GG	246	239	1.067	0.883, 1.290	0.508
8. rs2747652 (ESR1)	1-CC	1377	1372				20. rs858518 (SHBG)	1-TT	1693	1597			
(6/152437016)	2-CT	2479	2447	1.009	0.918, 1.109	0.849	(17/7533025)	2-TC	2412	2490	0.914	0.836, 0.999	**0.047**
	3-TT	1144	1181	0.965	0.863, 1.080	0.535		3-CC	895	913	0.925	0.823, 1.039	0.188
9. rs2077647 (ESR1)	1-AA	1383	1347				21. rs272428 (SHBG)	1-CC	1609	1523			
(6/152129077)	2-AG	2449	2589	0.921	0.838, 1.012	0.087	(5/179323119)	2-CT	2438	2442	0.945	0.863, 1.035	0.225
	3-GG	1168	1064	1.069	0.954, 1.198	0.242		3-TT	953	1035	0.872	0.778, 0.977	**0.017**
10. rs2175898 (ESR1)	1-AA	1350	1353				22. rs858524 (SHBG)	1-AA	1613	1725			
(6/152196952)	2-AG	2507	2457	0.846	0.768, 0.932	**0.001**	(17/7511287)	2-AG	2459	2393	1.099	1.005, 1.201	**0.037**
	3-GG	1143	1190	0.941	0.852, 1.040	0.238		3-GG	928	882	1.125	1.002, 1.264	**0.044**
11. rs9340799 (ESR1)	1-AA	2016	2107				23. rs2017591 (STS)	1-TT	1823	1760			
(6/152163381)	2-AG	2360	2302	1.071	0.984, 1.166	0.109	(X/7158114)	2-TC	2258	2437	0.895	0.819, 0.977	**0.012**
	3-GG	624	591	1.103	0.969, 1.257	0.133		3-CC	919	803	1.105	0.983, 1.242	0.094
12. rs1709182 (ESR1)	1-TT	1932	1988										
(6/152175357)	2-TC	2326	2341	1.022	0.938, 1.114	0.618							
	3-CC	742	671	1.138	1.006, 1.288	**0.038**							

a Data collected from literature [14].

b Data highlighted in bold text are statistically significant results.

c All the [Ch/position], i.e., [Chromosome no./Chromosome position], information is based on “Assembly GRCh37”.

d The contig information is shown in SNP no. (contig accession no.) as follows: SNP 1–2 (NT_011519.10); SNPs 3 (NT_010194.17); SNPs 4–15 (NT_025741.15); SNPs 16–19 (NT_033899.8); SNPs 20–22 (NT_010718.16); SNPs 23 (NT_167197.1).

e Values with *p* value<0.05 are highlighted in bold fonts.

Meanwhile, the 3-SNP barcodes are generated in a random way (step 2-2b) and then sorted by the order of the fitness values (step 3-3b). The result from step 3-3a is used to replace the worst of the top five SNP barcodes (step 3-4). Finally, the updated 3-SNP barcode population is ready for the PSO computation, in which the top five in amongst the 3-SNP barcodes (step 3-5) are determined. Now, the top five SNP barcodes can be used to start the generation of the next higher order SNP barcodes. The steps are described in detail by the annotated IPSO pseudo-code in the next two sections.

**Table 4:** The best estimated protective SNP combinations on the occurrence of breast cancer as determined by IPSO.

Number of combined SNPs(specific SNPs)	SNP genotypes	Control no./Case no.	Difference(specific SNPs)	Correct	Sen.+Spe.	PPV+NPV	Risk Ratio	Odds Ratio(*p value*)
2-SNP	others	3596/3770						0.84
SNPs(4-19)	1-1	1404/1230	174	0.48	0.97	0.96	0.88	(**<0.001** *)
3-SNP	others	4301/4429						0.79
SNPs(4-19-23)	1-1-2	699/571	128	0.49	0.97	0.94	0.82	(**<0.001** *)
4-SNP	Others	4644/4731						0.74
SNPs(4-9-19-23)	1-2-1-2	356/269	87	0.49	0.96	0.93	0.76	(**<0.001** *)
5-SNP	Others	4809/4864						0.70
SNPs(3-4-9-19-23)	2-1-2-1-2	191/136	55	0.50	0.99	0.91	0.71	(**0.002** *)
6-SNP	Others	4911/4946						0.60
SNPs(3-4-9-13-19-23)	2-1-2-2-1-2	89/54	35	0.50	0.99	0.88	0.61	(**0.004** *)
7-SNP	Others	4951/4972						0.57
SNPs(3-4-9-13-19-20-23)	2-1-2-2-1-2-2	49/28	21	0.50	1.00	0.87	0.57	(**0.022** *)
8-SNP	Others	4971/4983						0.59
SNPs(3-4-9-12-13-19-20-23)	2-1-2-2-2-1-2-2	29/17	12	0.50	1.00	0.87	0.59	(0.103)
9-SNP	Others	4986/4994						0.43
SNPs(3-4-9-12-13-14-19-20-23)	2-1-2-2-2-2-1-2-2	14/6	8	0.50	1.00	0.80	0.43	(0.115)
10-SNP	Others	4994/4999						0.17
SNPs(3-4-9-12-13-14-19-20-21-23)	2-1-2-2-2-2-1-2-3-2	6/1	5	0.50	1.00	0.64	0.17	(0.125)

* The SNP combinations on the occurrence of breast cancer are significantly different (*p value*<0.05). Sen.; Sensitivity; Spe., specificity; PPV, positive predictive value; NPV, negative predictive value. The meanings of the SNP and genotype numbers are provided in Table 3. For example, barcode SNPs (4-19)-genotype (1-1) is [rs3020314-CC]-[rs500760-AA]; SNPs (4, 19, 23) with genotype 1-1-2; [rs3020314-CC]-[rs500760-AA]-[rs2017591-TC].

#### Fitness function

In this study, the fitness value means are used to compute the difference between the case and control data from the selected SNP combinations. The focus lies on specific SNP combinations to obtain the highest fitness value, i.e., the maximum SNP combination difference between cases and controls. The concept uses the intersection of set theory to compute the difference between cases and controls. The intersection of two sets is the set that contains all elements of one of these sets that also belong to the other set, but no other elements. A high fitness value indicates the best combination of an SNP and genotypes. The relevant equation is shown below:
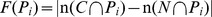
(3)where n represents the total number of elements in a set. *C* represents the total number of SNP interactions in the case group, and *N* represents the total number of SNP interactions in the control group. *P_i_* represents the *i*th particle. The fitness value definition can be divided into three steps. First, the total number of intersections of the case data set and the *i*th particle is calculated as n(*C*∩*P_i_*). Second, the total number of intersections of the control data set and the *i*th particle is calculated as n(*N*∩*P_i_*). Finally, Eq (3) is used to calculate the fitness value that is the difference between the intersection of the case and the particle and the intersection of the control and the particle. For example, *P = (SNP_1,2_, Genotype_2,1_)* it is used to compute the number matching the condition of the SNP and genotypes for the case and control in the breast cancer data. First, the number of controls for *SNP_1_* with genotype 2 and *SNP_2_* with genotype 1 is calculated. The number of cases independently matching *SNP_1_* with genotype 2 and *SNP_2_* with genotype 1 was 76 in the breast cancer data set. Second, the number of controls independently matching *SNP_1_* with genotype 2 and *SNP_2_* with genotype 1 is calculated as 141. According to Eq. (3), the fitness value is determined by subtracting 76 from 141, giving -65. If the fitness is negative, the absolute value is taken to obtain a fitness value of 65.

#### Identification of pbest and gbest

Each particle finds its personal best position (*pbest*) and the global best position (*gbest*) when moving. If the fitness value of a particle *P_i_* in the current iteration is better than the fitness value of *pbest* in the previous iteration, *pbest* is updated to that of *P_i_*. If the fitness value of particle *P_i_* in the current iteration is better than *gbest* in the previous iteration and is the best one in the current iteration, *gbest* is updated to that of *P_i_*. Each particle then adjusts its direction based on *pbest* and *gbest* in the following iteration.

As mentioned in Table 1, the pseudo-code for IPSO algorithm can collocate data with the adaptation procedure as mentioned above and generate the best SNP barcode for breast cancer prediction.

**Figure 2:**
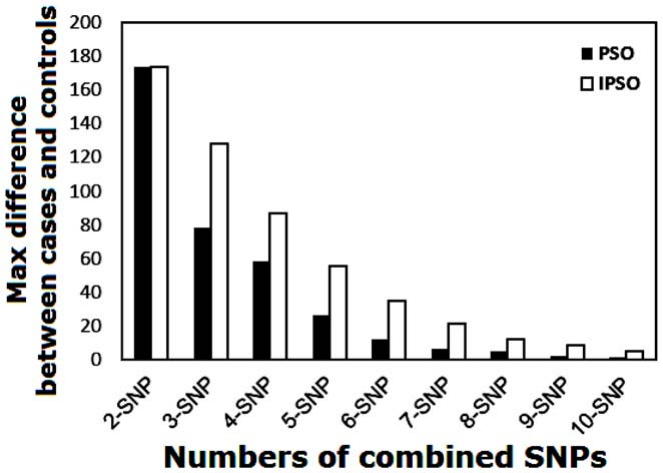
The maximum difference between cases and controls for PSO and IPSO on the best barcodes containing two to ten SNPs.

#### Parameter settings

The population size parameter was set to 50 (Figure 1, step 2-2b). The termination condition of the PSO is reached at a prespecified number of iterations (in our case, the number of iterations is 100) (Figure 1, step 3-5). The other parameters used in the PSO were *c*_1_ = *c*_2_ = 2. *V*_max_ was equal to (*X*_max_ – *X*_min_) and *V*_min_ was equal to – (*X*_max_ – *X*_min_). These parameters have been optimized by Kennedy and Eberhart [33].

#### Performance measurement and statistical analysis

We used five commonly used criteria to determine the performance [28].
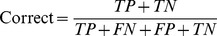
(4)

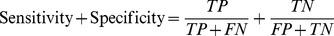
(5)

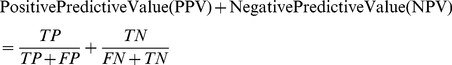
(6)

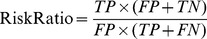
(7)

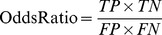
(8)


*TP*, *TN*, *FN*, and *FP* represent the number of true positives, true negatives, false negatives, and false positives, respectively. For statistics analysis with SPSS 13.0, the risk ratio (*RR*) and odds ratio (*OR*) are used to determine the best SNP barcode and quantitatively measure the breast cancer risk. The boxplots were analysed by SigmaPlot 9.0 (Systat Software, Inc.).

## Results

### Data Set Preparation

The data set for the steroid hormones and their signalling and metabolic pathways (96 SNPs for 8 genes) were obtained from the breast cancer association study in [14]. This data set only provides the genotype frequencies without the original raw data for the genotypes of each SNP. In our study, we simulated the genotype data based on the original frequencies of the data set. Using the simulated genotype data, susceptibility to breast cancer in terms of complex SNP-SNP interactions can be considered. However, it does not reflect the true distribution of those SNPs in cases and controls, and therefore results are not real. However, the original data involves different numbers of genotypes, and hence we had to perform normalization to make each genotype size the same in order to allow further analysis. Our simulated data was randomly generated and obeys the original genotype frequency in the entire data set; the simulated data is available at http://bioinfo.kmu.edu.tw/brca-steroid-96SNP.xlsx.

The normalization procedure is provided in the “pseudo-code for randomly generated data” as shown in Table 2. For example, we set the range size to a maximum range of 5000, and then calculate the amount of three genotypes in each SNP. The example of SNP_4_ (rs3020314) includes 4551 genotypes in the original data, which contain 2132 for CC, 1970 for CT, and 449 for TT, respectively (the step for pseudo-code 04). In each SNP, the percentage of each genotype is calculated, for the above instance, 2132/4551 (46.85%) for CC, 1970/4551 (43.29%) for CT, and 449/4551 (9.86%) for TT. Based on these percentages, the modified data for SNP_4_ is obtained by multiplication of the percentage with the amount of the entire data set, i.e., 46.85%×5000 = 2343 for CC, 43.28%×5000 = 2164 for CT and 9.86%×5000 = 493 for TT (the step for pseudo-code 05 to 11). The simulated data for SNP_4_ has thus been normalized to 5000 (2343+2164+493 = 5000). Accordingly, all original data are normalized to the same number in this manner.

**Figure 3:**
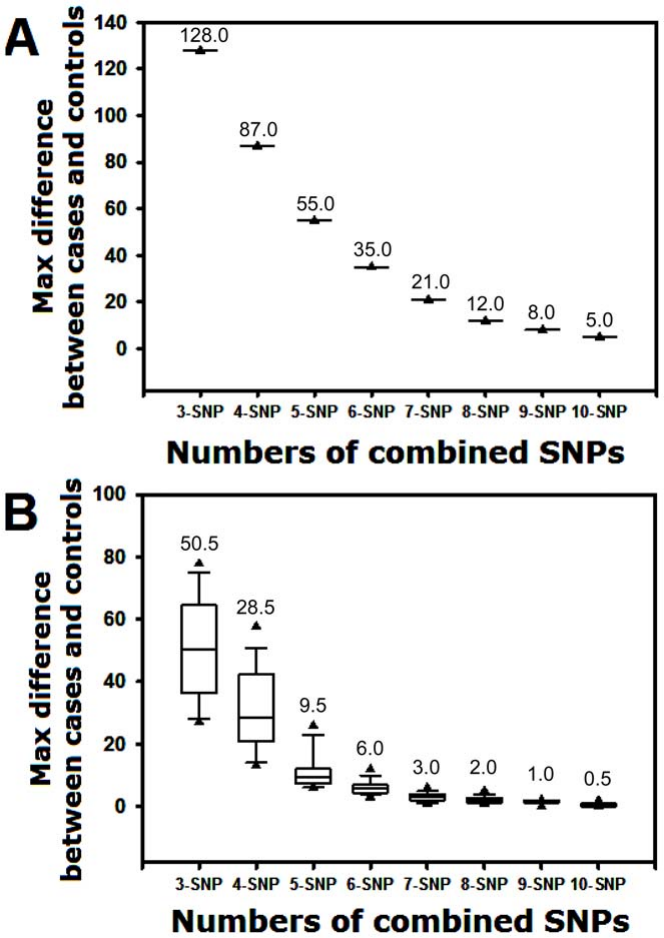
Boxplots displaying the extremes, the upper and lower quartiles, and the median of the maximum difference between cases and controls for (A) the IPSO algorithm and (B) the PSO algorithm on three to ten combined SNPs over 20 runs. The boundary of the box closest to zero indicates the 25th percentile, a line within the box marks the median, and the boundary of the box farthest from zero indicates the 75th percentile. Error bars above and below the boxes indicate the 90th and 10th percentiles, respectively. The triangle symbols indicate the 95th and 5th percentiles.

### Evaluation of Breast Cancer Susceptibility in 23 Separate SNPs from 6 Steroid Hormone Metabolisms and Signalling-related Genes

Based on our simulated data, Table 3 shows the performance (*OR* and 95% CI) for each SNP from 6 steroid hormone metabolisms and signalling-related genes (COMT, CYP19A1, ESR1, PGR, SHBG, and STS). Some SNPs (such as SNPs 4, 10, 12–15, 17, and 19–23 listed in Table 3) with certain genotypes display a statistically significant *OR* (*p*<0.05) for breast cancer; their *OR* values range from 1.268 to 0.846. The other SNPs show no statistically significant *OR* for breast cancer.

**Table 5:** The best estimated protective SNP combinations on the occurrence of breast cancer as determined by PSO.

Number of combined SNPs(specific SNPs)	SNP genotypes	Control no./Case no.	Difference(specific SNP)	Correct	Sen.+Spe.	PPV+NPV	Risk Ratio	Odds Ratio(*p value*)
2-SNP	others	3596/3770						0.84
SNPs(4-19)	1-1	1404/1230	174	0.48	0.97	0.96	0.88	(**<0.001** *)
3-SNP	others	4427/4505						0.85
SNPs(4-22-23)	1-2-2	573/495	78	0.49	0.98	0.96	0.86	(**0.013** *)
4-SNP	Others	4670/4728						0.81
SNPs(9-18-19-23)	2-2-1-2	330/272	58	0.49	0.99	0.95	0.82	(**0.016** *)
5-SNP	Others	4885/4911						0.77
SNPs(3-4-12-20-23)	2-1-1-2-2	115/89	26	0.50	1.00	0.93	0.77	(0.077)
6-SNP	Others	4950/4962						0.76
SNPs(12-15-17-19-21-22)	2-2-2-1-2-1	50/38	12	0.50	1.00	0.93	0.76	(0.239)
7-SNP	Others	4982/4988						0.67
SNPs(2-7-14-18-19-21-23)	2-2-1-2-1-1-2	18/12	6	0.50	1.00	0.90	0.67	(0.361)
8-SNP	Others	4990/4995						0.50
SNPs(9-10-11-13-17-20-21-23)	3-3-2-2-1-2-2-2	10/5	5	0.50	1.00	0.83	0.50	(0.301)
9-SNP	Others	4993/4995						0.71
SNPs(1-3-4-11-14-16-18-21-22)	2-2-2-1-2-2-1-1-1	7/5	2	0.50	1.00	0.92	0.71	(0.774)
10-SNP	Others	4998/4999						0.50
SNPs(1-3-5-9-10-13-18-20-21-22)	2-2-2-2-2-3-1-1-3-1	2/1	1	0.50	1.00	0.83	0.50	(1.000)

* The SNP combinations on the occurrence of breast cancer are significantly different (*p value*<0.05). The meanings of the SNP and genotype numbers are provided in Table 3.

### Identification of the SNP-SNP Interactions with Maximum Differences between Cases and Controls Using IPSO

Using the IPSO algorithm, the best SNP-SNP interaction is evaluated by the difference between cases and controls for all the SNP barcodes. After computation, the top five of the 2-SNP barcodes can be listed in order of the difference between cases and controls: SNPs (4-19)-genotype (1-1), SNPs (4-23)-genotype (1-2), SNPs (4-9)-genotype (1-2), SNPs (19-23)-genotype (1-2), and SNPs (9-23)-genotype (2-2). The differences in the number of cases and controls for these SNP barcodes are 174, 168, 158, 150, and 146, respectively (data not shown). In this study, as shown in Table 4, we only select the 2-SNP barcodes with a maximum difference, i.e., the best 1 of the 2-SNP barcode. Similarly, the n-SNP barcodes (n = 3 to 10) with maximum differences, i.e., the best for each n-SNP barcode, are also selected (left side of Table 4).

With the conservation of the top five results, we found that the best for n-SNP barcode contains the corresponding best (n-1)-barcode. For example, the 3-SNP barcode contains the 2-SNP barcode, i.e., SNPs (4-19-**23**)-genotypes (1-1-**2**) *vs.* SNPs (4-19)-genotypes (1-1), where the bold letters indicate the newly selected SNP. The 4-SNP barcode contains the 3-SNP barcode, i.e., SNPs (4-**9**-19-23)-genotypes (1-**2**-1-2) *vs.* SNPs (4-19-23)-genotypes (1-1-2).

### Prediction Scores of the Best IPSO-generated SNP Barcodes in Breast Cancer

The best n-SNP barcodes (n = 3 to 10) calculated by the IPSO algorithm are listed in Table 4 to calculate their five prediction scores, i.e., the correctness, sensitivity+specificity, PPV+NPV, *RR*, and *OR*, in order to evaluate the breast cancer susceptibility based on the IPSO-generated SNP barcodes. The sensitivity and specificity values of the respective best SNP barcodes are all higher than 0.96, suggesting that IPSO can identify the best SNP barcodes associated with breast cancer. The correctness and PPV+NPV values of the respective best SNP barcodes range from 0.48 to 0.50 and 0.64 to 0.96, respectively, and the *RR* and *OR* of the best SNP barcodes range from 0.88 to 0.17 and 0.84 to 0.17, respectively. The SNP barcodes involving two to seven SNPs show significantly decreasing *OR* values (*p*<0.05 to 0.001). Since the SNP barcodes listed in Table 4 show that the control numbers are greater than the case numbers, the SNP barcodes are regarded as protective SNP barcodes against breast cancer.

### Comparison between the Best IPSO-generated and PSO-generated SNP Barcodes in Breast Cancer

We compare IPSO with PSO for the reliability and the ability to identify SNP barcodes to support the advantage of the top-five strategy. The performances of the PSO and IPSO algorithms from 20 simulation runs (see supplement Table S1 and Table S2 for details) are compared by means of the best maximum difference between cases and controls as shown in Figure 2. To examine the performance in terms of the statistical differences between both algorithms, we performed the Wilcoxon Signed-Rank test and found that there were significant differences between cases and controls in n-SNP barcodes (n = 2 to 10) (Table S3).

The maximum differences for each SNP barcode generated by IPSO are higher than those of PSO, suggesting that the selection of the best protective SNP barcodes is more reliable in IPSO than in PSO. As shown in Figure 3, the median value results suggest that IPSO is more suitable for selecting the best SNP barcodes for breast cancer protection. Moreover, the interquartile ranges (25th to 75th) of the boxplot, as well as the 5th, 10th, 90th and 95th percentiles for each n-SNP barcode (n = 3 to 10), are more narrow in the IPSO algorithm (Figure 3A) than in the PSO algorithm (Figure 3B). These data suggest that the results of the PSO algorithm are more unstable. In contrast, the IPSO algorithm provides exact identification of the best SNP barcodes for breast cancer protection. Actually, the data in Figure 3A (IPSO) are all the same for each n-SNP (n = 3 to 10), i.e., 128, 87, 55, 35, 21, 12, 8, and 5 (Table 4). The best PSO-generated n-SNP barcodes with maximum differences between cases and controls are listed in Table 5. In the PSO algorithm the top five results are not conserved. Accordingly, the PSO-generated SNP barcodes conserve the selected SNPs to a lesser degree (Table 3). For example, only one SNP in the 2-SNP barcode shows up in the 3-SNP barcode, i.e., SNP 4 (rs3020314; Table 3), and only one SNP in the 3-SNP barcode shows up in the 4-SNP barcode, i.e., SNP 23 (rs2017591). Therefore, an order of influence on breast cancer is very difficult to establish from the SNPs in Table 3.

## Discussion

Many association studies of cancer focused on the analysis of risk genetic factors that influence common complex traits in terms of commonly occurring SNPs. However, the possible protective effects are also important for the prediction of cancer morbidity by SNPs. Here, we analyzed the contribution of 23 SNPs from six breast cancer related genes to generate the protective SNP barcodes in a case-control study of 5000 cases and 5000 controls with genotype data simulation.

The maximum difference information calculated by the IPSO algorithm can predict the relative strength of the impact of an SNP on breast cancer protection. For example, the difference between controls and cases for SNP barcode [SNPs (4-19)-genotype (1-1)] is higher than that of [SNPs (4-19-23)-genotype (1-1-2)], suggesting that SNP 4 and SNP 19 are more associated with breast cancer protection than SNP 23. Accordingly, an order of impact on breast cancer for the SNPs listed in Table 3 can be arranged: SNPs 4/19> SNP 23> SNP 9> SNP 3> SNP 13> SNP 20> SNP 12> SNP 14> SNP 21. In this simulated breast cancer association study, the IPSO-generated SNP barcodes involving two to seven SNPs and two to four SNPs show significantly decreasing *OR* values ranging from 0.84 to 0.57 (Table 4). In contrast, some individual SNPs with certain genotypes display statistically significant *OR* values ranging from 1.268 to 0.846 (Table 3).

Some SNPs may display different impacts on the protection of breast cancer in terms of the individual SNPs or the combinational SNPs. For example, some individual SNPs such as SNPs 3 and 9 are not significantly associated with breast cancer (Table 3), but the occurrence of 4- to 10-SNP combinations including SNPs 3 and 9 shows the significant association with breast cancer (Table 4). These data suggest that the association relationship for breast cancer may be ignored when the SNP interaction is of no concern.

A key issue of detecting SNP-SNP interactions in genome-wide case-control study is the computational efficiency. The computational complexity of IPSO algorithm is estimated by the objective function computation. If there are M number of iterations and N number of solutions (particles) in the population, then the objective function computation has O(MN) computational complexity. The effective feature of the top 5 strategy computation is only storing the top 5 solutions in each iteration. If there are K solutions in the archive, storing the solutions in the archive has O(M+K) computational complexity. If the archive and the iteration have the same numbers, the overall complexity of IPSO is O(MN+K).

Although the optimal parameters of PSO were demonstrated by Kennedy and Eberhart [33], we found that the parameter adjustments may promote better results even for large numbers of SNPs. Firstly, the population and iterations could adjust its size according the data size, in which the population size suggested setting from 50 to 100 and number of iterations suggested setting from 100 to 1000, i.e., it explores to better SNP barcodes with large difference between cases and controls, but the computational complexity of IPSO is also increased. Secondly, the *c*_1_ and *c*_2_ are acceleration constants that control how far a particle moves in a single generation, and they respectively control the exploitation and exploration ability in each search. In order to balance the exploitation and exploration, the *c*_1_ and *c*_2_ are suggested the same as 2.

Although we explored the benefit of IPSO algorithm for SNP interaction based on the simulated breast cancer study, the IPSO algorithm is not exclusively into breast cancer data and can be applied to other real data sets. After running these algorithms using another disease with the real dataset [18], e.g., osteoporosis, we found that the IPSO algorithm again showed better performance for selecting SNP barcodes in SNP-SNP interaction studies than the PSO algorithm (data not shown).

IPSO can overcome the limitations imposed on computational time for complex SNP interactions for GWAS because IPSO has the following advantages: 1) IPSO allows robust analysis of high-order SNP combinations for GWAS studies and generates the best SNP barcodes; 2) IPSO is an improved evolutionary algorithm without exhaustive search; 3) IPSO only needs two parameters for computation without complex settings; and 4) Its computational complexity is unaffected by the size of data sets.

In conclusion, we propose an improved PSO algorithm to perform a powerful breast cancer association analysis in terms of SNP-SNP interactions with 23 SNPs. Our strategy successfully improves on the performance of traditional PSO in terms of the reliability with a combination of more statistically significant SNPs associated with breast cancer protection. With the help of the IPSO algorithm, the best fitness of cases and controls can be identified. The algorithm can potentially be applied to identify complex SNP-SNP (gene-gene) interactions for different diseases, even in cases where a large number of SNPs is involved in genome-wide association studies.

## Supporting Information

Table S1 The estimated protective SNP combinations on the occurrence of breast cancer as determined by IPSO.
(PDF)

Table S2 The estimated protective SNP combinations on the occurrence of breast cancer as determined by PSO.
(PDF)

Table S3 Wilcoxon Signed-Rank test for IPSO and PSO.
(PDF)
