# Supplementary material for: An Improved PSO Algorithm for Generating Protective SNP Barcodes in Breast Cancer
Source: PLoS One. 2012 May 18;7(5):e37018. doi: 10.1371/journal.pone.0037018 (PMC3356401; doi:10.1371/journal.pone.0037018)
Supplement: Table S1 — The estimated protective SNP combinations on the occurrence of breast cancer as determined by IPSO. (PDF) [file pone.0037018.s001.pdf]

Table S1. The estimated protective SNP combinations on the occurrence of breast cancer as determined by IPSO

Test nos.	3-SNP	4-SNP	5-SNP	6-SNP	7-SNP	8-SNP	9-SNP	10-SNP
	Control no./ Case no. (Difference)							
1	699/571 (128)	356/269 (87)	191/136 (55)	89/54 (35)	49/28 (21)	29/17 (12)	14/6 (8)	6/1 (5)
2	699/571 (128)	356/269 (87)	191/136 (55)	89/54 (35)	49/28 (21)	29/17 (12)	14/6 (8)	6/1 (5)
3	699/571 (128)	356/269 (87)	191/136 (55)	89/54 (35)	49/28 (21)	29/17 (12)	14/6 (8)	6/1 (5)
4	699/571 (128)	356/269 (87)	191/136 (55)	89/54 (35)	49/28 (21)	29/17 (12)	14/6 (8)	6/1 (5)
5	699/571 (128)	356/269 (87)	191/136 (55)	89/54 (35)	49/28 (21)	29/17 (12)	14/6 (8)	6/1 (5)
6	699/571 (128)	356/269 (87)	191/136 (55)	89/54 (35)	49/28 (21)	29/17 (12)	14/6 (8)	6/1 (5)
7	699/571 (128)	356/269 (87)	191/136 (55)	89/54 (35)	49/28 (21)	29/17 (12)	14/6 (8)	6/1 (5)
8	699/571 (128)	356/269 (87)	191/136 (55)	89/54 (35)	49/28 (21)	29/17 (12)	14/6 (8)	6/1 (5)
9	699/571 (128)	356/269 (87)	191/136 (55)	89/54 (35)	49/28 (21)	29/17 (12)	14/6 (8)	6/1 (5)
10	699/571 (128)	356/269 (87)	191/136 (55)	89/54 (35)	49/28 (21)	29/17 (12)	14/6 (8)	6/1 (5)
11	699/571 (128)	356/269 (87)	191/136 (55)	89/54 (35)	49/28 (21)	29/17 (12)	14/6 (8)	6/1 (5)
12	699/571 (128)	356/269 (87)	191/136 (55)	89/54 (35)	49/28 (21)	29/17 (12)	14/6 (8)	6/1 (5)
13	699/571 (128)	356/269 (87)	191/136 (55)	89/54 (35)	49/28 (21)	29/17 (12)	14/6 (8)	6/1 (5)
14	699/571 (128)	356/269 (87)	191/136 (55)	89/54 (35)	49/28 (21)	29/17 (12)	14/6 (8)	6/1 (5)
15	699/571 (128)	356/269 (87)	191/136 (55)	89/54 (35)	49/28 (21)	29/17 (12)	14/6 (8)	6/1 (5)
16	699/571 (128)	356/269 (87)	191/136 (55)	89/54 (35)	49/28 (21)	29/17 (12)	14/6 (8)	6/1 (5)
17	699/571 (128)	356/269 (87)	191/136 (55)	89/54 (35)	49/28 (21)	29/17 (12)	14/6 (8)	6/1 (5)
18	699/571 (128)	356/269 (87)	191/136 (55)	89/54 (35)	49/28 (21)	29/17 (12)	14/6 (8)	6/1 (5)
19	699/571 (128)	356/269 (87)	191/136 (55)	89/54 (35)	49/28 (21)	29/17 (12)	14/6 (8)	6/1 (5)
20	699/571 (128)	356/269 (87)	191/136 (55)	89/54 (35)	49/28 (21)	29/17 (12)	14/6 (8)	6/1 (5)
