# Supplementary material for: An Improved PSO Algorithm for Generating Protective SNP Barcodes in Breast Cancer
Source: PLoS One. 2012 May 18;7(5):e37018. doi: 10.1371/journal.pone.0037018 (PMC3356401; doi:10.1371/journal.pone.0037018)
Supplement: Table S2 — The estimated protective SNP combinations on the occurrence of breast cancer as determined by PSO. (PDF) [file pone.0037018.s002.pdf]

Table S2. The estimated protective SNP combinations on the occurrence of breast cancer as determined by PSO

Test nos.	3-SNP	4-SNP	5-SNP	6-SNP	7-SNP	8-SNP	9-SNP	10-SNP
	Control no./ Case no. (Difference)							
1	516/441 (75)	180/147 (33)	42/35 (7)	17/13 (4)	18/12 (6)	10/7 (3)	3/1 (2)	N.E.
2	352/324 (28)	217/187 (30)	51/40 (11)	41/36 (5)	8/5 (3)	3/2 (1)	N.E	N.E.
3	492/452 (40)	330/272 (58)	134/112 (22)	23/18 (5)	15/11 (4)	3/2 (1)	3/2 (1)	2/1 (1)
4	482/425 (57)	320/271 (49)	96/87 (9)	29/26 (3)	N.E.	10/5 (5)	3/1 (2)	N.E.
5	476/425 (51)	269/242 (27)	68/56 (12)	48/38 (10)	2/1 (1)	3/1 (2)	5/3 (2)	N.E.
6	428/395 (33)	251/218 (33)	102/90 (12)	27/21 (6)	N.E.	2/1 (1)	N.E	2/1 (1)
7	578/528 (50)	178/155 (23)	55/47 (8)	69/64 (5)	23/19 (4)	3/2 (1)	N.E	N.E.
8	204/175 (29)	299/254 (45)	46/40 (6)	18/12 (6)	10/8 (2)	5/3 (2)	3/2 (1)	N.E.
9	397/370 (27)	83/69 (14)	73/66 (7)	15/11 (4)	N.E.	2/1 (1)	N.E	2/1 (1)
10	564/519 (45)	266/233 (33)	164/141 (23)	26/19 (7)	16/11 (5)	7/4 (3)	N.E	N.E.
11	394/327 (67)	172/145 (27)	62/55 (7)	39/32 (7)	12/8 (4)	5/3 (2)	N.E	3/2 (1)
12	397/351 (46)	139/119 (20)	52/46 (6)	9/4 (5)	22/20 (2)	16/12 (4)	2/1 (1)	N.E.
13	503/454 (49)	287/239 (48)	77/68 (9)	22/15 (7)	8/6 (2)	3/2 (1)	N.E.	N.E.
14	654/581 (73)	223/188 (35)	72/60 (12)	13/9 (4)	5/2 (3)	16/12 (4)	N.E.	N.E.
15	404/351 (53)	225/199 (26)	85/76 (9)	21/15 (6)	13/9 (4)	N.E.	N.E.	N.E.
16	476/425 (51)	269/242 (27)	68/56 (12)	48/38 (10)	2/1 (1)	3/1 (2)	5/3 (2)	N.E.
17	414/360 (54)	167/150 (17)	37/27 (10)	50/38 (12)	15/12 (3)	6/4 (2)	4/3 (1)	N.E.
18	565/490 (75)	66/53 (13)	115/89 (26)	53/44 (9)	28/24 (4)	N.E.	N.E.	N.E.
19	241/206 (35)	353/302 (51)	86/71 (15)	21/17 (4)	9/6 (3)	5/4 (1)	7/5 (2)	N.E.
20	573/495 (78)	172/158 (14)	75/66 (9)	38/31 (7)	3/1 (2)	3/1 (2)	N.E.	N.E.

N.E.: Not estimable.
