# Supplementary material for: An Improved PSO Algorithm for Generating Protective SNP Barcodes in Breast Cancer
Source: PLoS One. 2012 May 18;7(5):e37018. doi: 10.1371/journal.pone.0037018 (PMC3356401; doi:10.1371/journal.pone.0037018)
Supplement: Table S3 — Wilcoxon Signed-Rank test for IPSO and PSO. (PDF) [file pone.0037018.s003.pdf]

Table S3. Wilcoxon Signed-Rank test for IPSO and PSO

	R^+	R^-	$R^=$	*p* value
IPSO *v.s.* PSO	8	0	1	0. 012

R^+: The number of differences between cases and controls in IPSO is bigger than in PSO.

R^-: The number of differences between cases and controls in IPSO is smaller than in PSO.

$R^=$: The number of differences between cases and controls in IPSO is equal to PSO.
